# Mandatory research projects during medical specialist training in Australia and New Zealand: a survey of trainees’ experiences and reports

**DOI:** 10.5694/mja2.52611

**Published:** 2025-02-25

**Authors:** Paulina Stehlik, Caitlyn Withers, Rachel C Bourke, Adrian G Barnett, Caitlin Brandenburg, Christy Noble, Alexandra Bannach‐Brown, Gerben B Keijzers, Ian A Scott, Paul P Glasziou, Emma C Veysey, Sharon Mickan, Mark Morgan, Hitesh Joshi, Kirsty Forrest, Thomas G Campbell, David A Henry

**Affiliations:** ^1^ Griffith University Gold Coast QLD; ^2^ Bond University Gold Coast QLD; ^3^ Gold Coast Hospital and Health Service Gold Coast QLD; ^4^ Institute of Health and Biomedical Innovation Queensland University of Technology Brisbane QLD; ^5^ The University of Queensland Brisbane QLD; ^6^ QUEST Center for Responsible Research Berlin Institute of Health at the Charité University Medical Hospital Berlin Germany; ^7^ Princess Alexandra Hospital Brisbane QLD; ^8^ Institute for Evidence‐Based Practice Bond University Gold Coast QLD; ^9^ St Vincent's Hospital Melbourne Melbourne VIC; ^10^ The Prince Charles Hospital Brisbane QLD; ^11^ Sunshine Coast University Hospital Kawana Waters QLD; ^12^ Clinical Trials Centre University of the Sunshine Coast Buderim QLD

**Keywords:** Research design

## Abstract

**Objective:**

To determine how many specialist trainees are required to conduct research projects, how they conduct these studies, and their views on the value of these activities; to assess the design and reporting quality of their research reports.

**Study design:**

Online, anonymous survey.

**Setting, participants:**

Current and recent trainees (past five years) at Australian and New Zealand specialist colleges, recruited through eleven colleges and snowballing; survey was available 31 March – 31 December 2021.

**Main outcome measures:**

Whether trainees were required to conduct research as part of specialty training; how they conducted their projects; the skills mix of the project team and access to relevant expertise and supervision; trainee views on mandatory research during specialty training; research engagement after training. Respondents were invited to submit project reports for reporting and methodological quality evaluation.

**Results:**

A total of 371 people commenced the survey; 361 respondents provided answers about mandatory research projects during specialist training, including 311 (86%) who had been required to complete projects. Seventy‐six of 177 people who had completed projects (43%) provided information about 92 projects and submitted 34 project reports for evaluation. Thirty‐eight projects (41%) investigated questions developed by the trainees alone; in 48 cases (52%) trainees had planned their projects with little outside input; of the 69 study protocols developed (75% of projects), 60 were developed by the trainees. The median proportion of time devoted to the research project exceeded 50% for trainees in ten of twelve colleges. Respondents typically worked in non‐collaborative teams, restricted to members of their own specialty, and additional expertise was limited to statisticians, allied health professionals, and nurses. Eighty‐seven of 174 participants who had completed projects (50%) felt that doing so was very or moderately important for their clinical careers; 36 of 67 respondents (54%) supported the requirement for scholarly projects during specialty training; 33 of 61 respondents (54%) had participated in research after completing training, and 44 (72%) had considered doing so. Twenty‐five of 34 available reports had been published; in 27 assessable reports, methods and results reporting was generally poor, and the risk of bias moderate to high in all but three. Participants criticised using their own time for projects and their potentially low quality results.

**Conclusion:**

For trainees who undertake specialty training, the time commitment and poor quality research associated with mandatory research projects were frequently concerns. Medical colleges should focus on research training tailored to individual career aspirations and training needs.

**The known**: Most Australian and New Zealand medical specialty colleges require trainees to undertake projects to develop research skills.

**The new**: In an anonymous survey, recent specialist trainees expressed concerns about the support available for planning and undertaking mandatory research projects, the time commitment required, and the poor quality of outcomes. Views on the quality of the research experience itself were mixed.

**The implications**: Requiring all specialist trainees to undertake projects yields inconsistent experiences and results. A more flexible approach, focused on research translation and participation in collaborative research, would recognise differences in career aspirations and training needs.

Medical specialty training colleges in North America,^1^, ^2^ Africa,^3^ China,^4^ the United Kingdom,^5^ Europe,^6^ and Australia^7^, ^8^ often require doctors to undertake research to earn professional qualifications. We have reported that 55 of 58 reviewed Australian college training programs require doctors to conduct and publish their own projects rather than develop research skills under expert supervision.^7^ This approach encourages rushed, poor quality, small scale projects, and trainees may not learn how high quality research contributes to patient care.^9^ The authors of a review of ten United Kingdom surgical programs similarly questioned the quality of the research output and trainee experiences.^5^

This approach to developing research skills has been criticised,^10^, ^11^, ^12^ but trainee research experiences and output have not been investigated in detail. We therefore surveyed medical specialty trainees in Australia and New Zealand about research activities as college training requirements, to establish how many trainees are required to conduct research projects, how they conducted the studies, and their general views on the value of these activities. We also assessed the design and reporting quality of their research reports.

## Methods

We conducted an anonymous survey of current and recent Australian or New Zealand medical specialty trainees in 2021. The recruitment materials, survey text, and analytic code (including packages) are available on the OFS website (https://osf.io/346xe). We report our study according to the CHERRIES guidelines for electronic surveys.^13^

### Eligibility and recruitment

We recruited people who were completing or had recently completed (within the preceding five years) specialty training programs at accredited Australian and New Zealand specialty training colleges. We cooperated with eleven medical specialty colleges (see Acknowledgements) to disseminate information about the survey, including a direct link to the survey, in newsletters and by email; we also directly emailed potential participants known to the investigators, and publicised the survey in slides at conferences and forums and in social media posts. Potential participants were also encouraged to share the invitation with eligible colleagues. Invitations were sent during 31 March – 17 September; the online survey was available until 31 December 2021 (further details: Supporting Information, part 1). As responses were anonymous, we could not track which recruitment method led to survey participation or calculate a response rate; we report the numbers of people who started the survey and of those who answered each question.

### Survey content

The survey comprised a participant eligibility check and three sections: the main survey (developed by the investigators) and two optional sections based on validated instruments (Supporting Information, part 2). We used a secure survey platform (Qualtrics), using survey logic to guide respondents through the survey according to their responses.

The survey was developed by a core group of authors (PS, CB, CN, DH), informed by published literature on problem areas in research^14^ and how best to support trainee research in the workplace.^15^ It was tested for face and content validity by the entire author group, which included people with expertise in medical education, clinical research, meta‐research, and evidence‐based practice, and people from several medical specialties. The survey was piloted with research team members, some of whom would have been eligible for survey participation or were trainee supervisors.

All questions in section 1 of the survey were mandatory, apart from the project file upload; it was about ten pages long (depending on responses). Participants were asked when and where they completed their most recent specialty training, their views on conducting research during specialty training, and how many projects they had completed. We defined a project as any project‐type work required by their college as part of specialty training, including primary research, secondary research (eg, systematic reviews), audits, and quality improvement projects.

For each project, we asked respondents how they formulated their research question, whether they undertook a literature review or developed a protocol before commencing the project, the skills mix of the project team, access to relevant expertise and supervision, and whether consumers (people with lived experience of the health question or topic investigated) were involved,^16^ the publication status of the project, and whether they believed that their research findings would be useful. We also asked about their satisfaction with the overall experience, skills development opportunities, and research engagement after training.

In sections 2 and 3 of the survey, we asked trainees to complete two validated questionnaires that assessed their research experience: the Postgraduate Research Experience Questionnaire (PREQ)^17^ and the WReN Spider instrument,^18^ each about one page long. Following pilot testing feedback and a desire to reduce the burden on trainees, these sections were not mandatory. As only ten participants completed these sections, we do not discuss these sections further in this article.

At the end of the survey, participants were asked if they wished to participate in in‐depth interviews about their experiences; this component of the study is reported elsewhere.^19^

### Quality assessment of research reports

We could not obtain trainee project reports directly from the colleges, as some colleges do not archive submitted reports and others require trainee consent to release reports. We therefore asked participants to upload a copy of the manuscript they submitted to the college or to provide a citation for a published article based on their project. We assessed whether the research question was clear, a study rationale was provided, the published literature had been adequately considered, and a sample size calculation was provided (if relevant). Depending on the study type, we appraised the quality of reporting according to EQUATOR guidelines (https://www.equator‐network.org/reporting‐guidelines) and that of study methods using appropriate critical appraisal tools (Supporting Information, part 3).

### Sample size

As we did not test a hypothesis, a formal sample size calculation was not performed.^20^ Assuming an acceptable margin of precision of 10% for standard prevalence estimates, and a worst‐case rate of completed and uploaded research projects of 20%,^21^ we estimated a sample size of around 480 responses would yield 96 completed research projects for our analysis.

### Statistical analysis

We summarise responses as numbers and proportions of responses for participants who answered at least one demographic data question. As we used survey logic, the number of eligible participants differed by question according to their previous responses. We did not conduct sensitivity analyses or adjust for the unrepresentativeness of our sample. Data analysis and visualisation were conducted in Python 3.10.9 and R 3.6.1 (R Foundation for Statistical Computing), using Jupyter notebooks.^22^

Free‐text survey responses (questions 13, 14b, 14d, 15) were subjected to qualitative content analysis, in which core meaning is derived from the text and grouped into themes,^23^ by an experienced qualitative research assistant using Microsoft Word. Themes were discussed by research team members with content and qualitative research expertise (authors PS, CB).

### Ethics approval

The study was approved by the Bond University Human Research Ethics Committee (PS00149).

## Results

Of the 426 eligible participants who commenced the survey, 371 people (87%) completed at least one demographic question (Box 1); the median time for survey completion was 5.3 minutes (interquartile range, 3.0–10.4 min). Of those who responded to the corresponding questions, 224 participants were women (61%) and 237 were currently in training (64%); 77 of 133 participants who had completed training (58%) had done so in the preceding two years. Training had been undertaken or was being undertaken in urban centres by 308 participants (84%) (Box 2). The 371 participants included people from all but one of the sixteen medical specialty colleges in Australia (314 participants) and New Zealand (48 participants). The number of respondents from Queensland, the investigators’ home state (102, 27%) was larger than its population proportion (Supporting Information, table 2).

Box 1 **Summary of selection and participation of current and recent Australian and New Zealand medical specialty trainees for our 2021 mandatory research project survey**

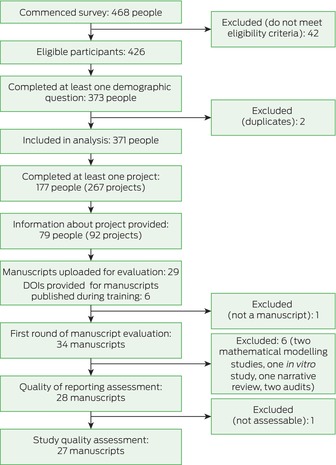

Box 2 Demographic characteristics and research project intentions of the participants in our 2021 medical specialty trainee mandatory research project survey*

**Table:** 

Characteristic	Participants
Gender	369/371 (99%)
Female	224 (61%)
Male	137 (37%)
Prefer not to say	7 (2%)
Non‐binary	1 (< 1%)
Completed training	370/37 (100%)
No	237 (64%)
Post training in past two years	77 (21%)
Post training more than two years ago	56 (15%)
Country (most recent specialist training)	365/371 (98%)
Australia	314 (86%)
New Zealand	48 (13%)
Other	3 (1%)
Residential location	365/371 (98%)
Urban	308 (84%)
Regional	42 (12%)
Rural/remote	15 (4%)
Completed or completing projects	361/371 (97%)
Yes	177 (49%)
In progress	76 (21%)
I plan to	58 (16%)
No	50 (14%)
Reason for not completing a project	47/50 (94%)
It was not required	20 (43%)
I had recognition of prior learning	6 (13%)
I completed a PhD instead	0
I completed a research masters degree instead	3 (6%)
I completed approved coursework instead	13 (28%)
Other	5 (11%)

* For each category the total number of respondents and the number of eligible respondents is provided; within categories, the proportions are based on the total number of responses for the category.

### Research projects

Of the 361 respondents who answered the first question about projects, 311 (86%) had completed, were completing, or were planning to complete research projects. Of 47 respondents who provided reasons for not undertaking projects, 20 reported that it was not required by their colleges, and 13 had instead completed approved coursework (Box 2). One‐hundred and seventy‐four (98%) respondents who had completed projects provided information on the number of projects they were required to complete. Forty‐eight (27%) reported completing more than one project, meaning that a total of 267 projects had been conducted by 174 trainees in our survey.

### Study provenance

Of 177 trainees who had completed projects, 79 (45%) provided further information about a total of 92 projects. Thirty‐eight projects (41%) investigated questions developed by the trainees alone, and 35 investigated questions that arose during clinical discussions (38%); fourteen projects (15%) formed part of ongoing research projects. Trainees had planned their projects with little input from other people in 48 cases (52%) and with significant input from others in 35 cases (38%); protocols had already been developed for nine studies (10%) (Box 3).

Box 3 Characteristics of the research projects and research teams in the projects undertaken by the participants in our 2021 medical specialty trainee mandatory research project survey*

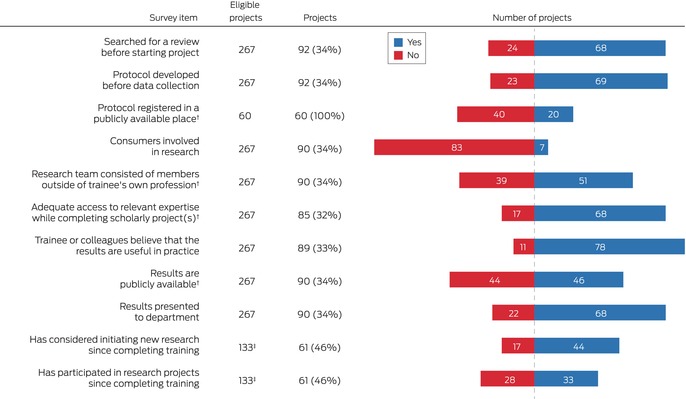

* For each item, the total number of respondents and the number of eligible respondents are provided. The complete data underlying this figure are provided in the Supporting Information, table 3.

† Multiple possible “yes” responses are pooled as a single number. For “protocol registered in a publicly available space”, the denominator is the number of trainee‐developed protocols.

‡ Number of participants, not projects.

Of the 69 study protocols developed (75% of projects), 60 were developed by the trainees, 20 of which were publicly available, including eleven in journals and seven in protocol registries. Sixty‐eight trainees (74%) searched for relevant systematic reviews of the literature before starting their projects (Box 3).

### Project support and collaboration

Trainees reported low levels of interdisciplinary and interprofessional collaboration; 39 of 90 project teams (43%) comprised only members of the trainee's specialty, and 40 project teams (44%) included people from only one other profession. Apart from members of their own profession, project teams most frequently included a statistician (21 cases), allied health professional (ten cases), or nurse (ten cases) (Supporting Information, table 3). Sixty‐eight of 85 respondents (80%) reported obtaining adequate expert support, most frequently clinical expertise (45 respondents), library services (22 respondents), and study design or measurement expertise (17 respondents). Seven of 90 projects (8%) involved participation by consumers (Box 3). Fifty‐seven of 85 respondents (67%) reported that they received adequate support from their supervisor for most projects (Box 4). The median proportion of time devoted to the research project exceeded 50% for trainees in ten of the twelve specialty colleges (Supporting Information, table 4).

Box 4 Views of medical specialty trainees undertaking mandatory research projects on their project experiences and the value of their projects*

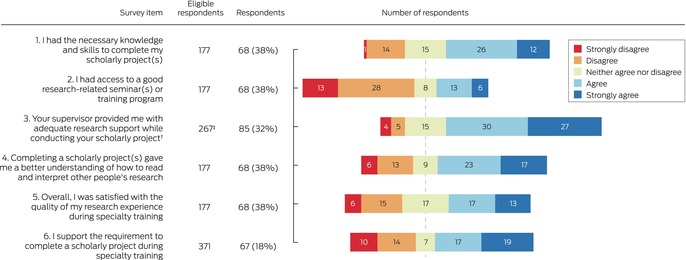

* For each item, the total number of respondents and the number of eligible respondents are provided.

† Four participants did not have supervisors.

‡ Number of projects, not participants.

### Perceived value of the research findings and dissemination of results

A publicly available research report was available for 46 of 90 projects (51%) (Box 3). Of the 45 studies published in journals, the trainee was the first author in 37 cases (88%); 33 (37%) had been published by the end of training. Seventy‐eight of 92 participants (88%) believed that their project findings would be useful in practice, and 81 of 90 (90%) felt confident about applying them in practice (Supporting Information, table 3).

### Respondents’ views on mandatory research projects

Eighty‐seven of 174 participants who had completed projects (50%) felt that doing so was very or moderately important for their clinical career (Supporting Information, table 3), and 40 of 68 respondents (59%) felt that completing a research project improved their ability to read and interpret research; 36 of 67 respondents (54%) supported the requirement for a scholarly project during specialty training (Box 4).

A total of 263 survey participants provided responses to the free‐text component of the survey. Sixty‐five participants mentioned the time required to do the research was unreasonable given clinical workloads and time away from family life and other activities, 51 felt that mandatory projects contributed to poor quality research, and 21 described them as “tick box” activities. Thirty‐nine participants described a lack of structured support in the training program, 36 felt the projects were a waste of time or not relevant to their career objectives, and 28 that there were better ways to learn evidence‐based practice or research skills. Twenty‐nine participants stated that research should be optional rather than mandatory, 44 that mandatory projects were important for develop skills beyond those related to research, 18 that it improved their evidence‐based practice skills, and 14 that they improved clinical practice (Box 5).

Box 5 **Summary of topics discussed in the free‐text responses by 263 participants in our 2021 medical specialty trainee mandatory research project survey**

**Table:** 

Theme	Respondents	Illustrative quotes
**Why trainees supported/opposed mandatory projects**		
Important to skills development including Evidence Based Medicine; improves practice; is important	76	Completion of this task is a good test of organisation, prioritisation irrespective of the research component. All medical staff should be able to critical[ly] appraise literature *(strongly supports mandatory projects)*. (participant 101) I am not personally strongly interested in pursuing research as part of my career but recognise that it is unavoidable in modern medicine and required as part of any job application *(moderately supports mandatory projects)*. (participant 17) Think it has some value for learning and rounding of a physician's skills *(moderately supports mandatory projects)*. (participant 324)
Contribution to research waste; tick box activity	72	To require true research for entrance or progression does nothing more than produce rubbish research *(neither supports nor opposes mandatory projects)*. (participant 91) I think in general mandatory research requirement to produce “papers” contribute to a large bubble of generally irrelevant papers which adds to a constant background of research noise that doesn't actually change practice *(moderately opposes mandatory projects)*. (participant 93) There is already an abundance of very questionable registrar level “research” diluting the pool of genuine, high quality, and clinically useful publications that are available. Cynically completing a research project because you are force[d] to do so does not benefit the individual or the profession, rather the opposite *(strongly opposes mandatory projects)*. (participant 13)
Time; unreasonable time requirements; time away from life; other prioritises	65	While knowing how to publish is a good thing for trainees, the practical experience of it is prohibitive and can delay completion of your training *(moderately supports mandatory projects)*. (participant 30) Whilst I strongly support research in general and feel the experience is beneficial personally I feel the requirement to carry out compulsory, time‐consuming research unpaid and with no allocated time whilst working more than full time and completing other training requirements and attending to family etc is unethical and needs to be reconsidered by all colleges *(moderately opposes mandatory projects)*. (participant 126)
No structural support	39	Almost the entire project is done in my spare time, this ended up being hundreds of hours … there is no access to any kind of research resources by the college, other than a handful of PDFs of previous projects on the website. It's a great idea, but as a trainee, I am tired of being forced to spend my spare time outside of work (when I should be relaxing/having a family/doing hobbies) devoted to mandatory training that is not supported by the college. We are stuck doing boring projects … on our own time, and end up with the worst of both worlds *(strongly supports mandatory projects)*. (participant 109) I do think research experience is important, but more support and guidance should be provided by colleges to meet their expectations. I had a disinterested supervisor (who I had to find myself) and a statistician who went on holiday for 5 months without telling me! It was a nightmare *(moderately supports mandatory projects)*. (participant 164) Without formalised and adequate oversight by knowledgeable staff, the quality of such endeavours is often poor … both projects were completed with essentially no outside input/help, so I can't really speak for the statistical quality or relevance of either *(neither supports nor opposes mandatory projects)*. (participant 85) Not enough support, guidance or time provided for project work. It is extremely difficult to find time to complete your project as well as an appropriate supervisor with the time, interest and experience in research *(strongly opposes mandatory projects)*. (participant 128)
Not relevant; waste of time	36	Most of us won't end up in research roles so while I feel that it's imperative that we know how to effectively interpret evidence, I don't think it's a great use of time to mandate research projects for trainees that don't have a particular interest in that area *(neither supports nor opposes mandatory projects)*. (participant 148) Seems a waste of time on the whole. Most of the “research” done as compulsory research for training isn't proper research, contributes little if anything to the field and doesn't teach the person doing it anything about real research (I say this having done proper research prior to medicine) *(moderately opposes mandatory projects)*. (participant 56) It is unnecessary for clinical work. Medical research should be its own specialty. There is so much crap research that is performed for the sake of it. It is a waste of time *(strongly opposes mandatory projects)*. (participant 42)
Optional; not mandatory	29	I think completing a scholarly project teaches essential skills in evidence‐based medicine and critical appraisal. However, I also acknowledge that not all doctors are interested in research and I don't think it should be made compulsory *(moderately supports mandatory projects)*. (participant 59) I can see some virtue to this, but the implication that every specialist has to be a researcher is invalid. Additionally, the requirement to conduct your own study and be first author (as opposed to participating in a multicentre study) excludes a lot of good experience and encourages poor practices *(neither supports nor opposes mandatory projects)*. (participant 34)
Better ways to learn research skills (including evidence‐based medicine)	28	There are other ways of developing research skills, particularly for those who have little or no interest in an academic path. For example, journal clubs, unit/departmental meetings *(neither supports nor opposes mandatory projects)*. (participant 1) It is valuable to participate in research though and to learn the finer points and have better understanding of the process. It would perhaps be more valuable to assess the quality of the project participated in and the contribution rather than the first author status *(moderately opposes mandatory projects)*. (participant 10) The skill in interpreting research is much better taught in an academic environment rather than forcing people without any background in research to complete often low quality research in an unsupported manner *(strongly opposes mandatory projects)*. (participant 58)
Project‐related problems (moving hospital, bureaucracy)	9	I think it is very hard to complete the research project when the requirements of training mean short contracts and constantly moving hospitals and states. Without a longer term engagement with one centre it is hard to be involved in meaningful research *(moderately opposes mandatory projects)*. (participant 55)
**Reasons trainees conducted research after their training**		
Have time or interest in research; believe that research is important; opportunities provided	41	I am in a role which allows non‐clinical time to achieve these goals. (participant 221) Because I am still passionate about [my research area] and it is an important part of my job. (participant 85)
Another training program	9	Had to do [a project] during my own subspecialty fellowship. (participant 173)
Supporting others/trainees	5	I'm involved in other trainees’ projects due to my skills. (participant 16)
**Reasons trainees did not conduct research after their training**		
Other prioritises; no time; not interested	24	I enjoy research, but again, very difficult to fit in whilst working full time. This is particularly true as a consultant. (participant 357)
No opportunities, support; interested, but …	13	Lack of funding or pathways to continue research. (participant 51)

Forty‐four of 61 respondents (72%) who had completed their specialty training had considered initiating new research since completing their training, and 33 of 61 (54%) had participated in research since completing training (Box 3). Fifty‐six participants provided free‐text responses regarding these aspects. Thirty‐six commented they now had more time and interest to participate in research, while 21 said they had no time for research (Box 5).

### Research report quality

Twenty‐four respondents uploaded 34 research reports: 25 journal articles, eight unpublished reports, and one poster (Supporting Information, table 5); 28 were assessed for quality (applicable standardised instruments were not available for the study types in six). Overall, the introduction and discussion sections were well reported, the methods and results less so (Supporting Information, figure 1). The risk of bias was moderate to high for 24 studies and low for three; one study was so poorly described that the risk of bias could not be assessed (Supporting Information, figure 2).

## Discussion

About 86% of the current and recent trainees who participated in our survey were required to complete research projects as part of specialty training, reflecting the requirements of most Australian and New Zealand colleges.^8^ About half of the respondents were solely responsible for developing the research questions, designing the projects, and developing study protocols, and few projects formed part of existing studies. In fourteen of sixteen specialties, at least half the project time required was their own time, which is recognised as a barrier to clinicians engaging meaningfully in research.^16^, ^24^ The exceptions were trainees at the Royal Australian College of General Practitioners (RACGP), where twenty trainees each year can conduct projects with protected time, and trainees at the Royal College of Pathologists of Australia, for whom the median research proportion undertaken during work time was 90% (IQR, 7–100%; four respondents). Although 57 of 85 trainees (67%) reported adequate support by project supervisors, respondents typically worked in non‐collaborative teams, often restricted to members of their own specialty, with access to additional expertise limited to statisticians, allied health professionals, and nurses. This finding may reflect a lack of research opportunities and resources, or the view that medical specialists should learn by leading research, rather than undertaking it in broad collaborations.

Some of the responses regarding projects were positive. Sixty‐eight of 92 respondents searched for relevant systematic reviews before starting projects (74%), 69 drafted research protocols (75%), and twenty of sixty registered protocols developed by trainees were publicly available. Forty‐two of the 92 respondents had published their project reports as journal articles, including 33 before they had completed training; 78 of 92 respondents (88%) thought their project findings were likely to be useful in their clinical practice, and 87 of 174 participants who had completed projects (50%) felt that the research experience was important for their career. These positive aspects were, however, offset by the results of the quality assessment of the uploaded reports; the risk of bias was high for 24 of 27 assessed studies, and the reporting of methods and results was often inadequate. Similar findings regarding articles by practising clinicians have been reported by other studies.^25^

Negative aspects of mandatory projects noted by respondents included the need to conduct projects in their own time, competing with family commitments; a lack of structured support; and concerns their projects were tick box exercises that yielded unhelpful research findings. Respondents also commented that learning how to apply research evidence in practice would be preferable to mandatory projects.

Our findings support those of other surveys. A study of Australasian College for Emergency Medicine (ACEM) trainees identified time and skills barriers to conducting research, and that learning outcomes were more consistently achieved with coursework than with scholarly projects.^21^ A 2020 survey of Royal Australian and New Zealand College of Radiologists trainees found significant proportions of unpublished research, limited access to statistics support, and mixed satisfaction with mandatory research.^26^ Surveys of overseas trainees regarding mandatory project requirements have yielded similar findings.^27^, ^28^

Most people believe that medical practitioners should be competent in translating research findings into practice; however, requiring every trainee to undertake a research project to learn these skills is not efficient.^29^ This view has been adopted by the RACGP^30^ and ACEM,^11^ as well as by some medical training programs overseas.^31^

Our survey findings suggest that the emphasis on individual research projects and authorship should change. The current approach assumes that all medical trainees aspire to be research leaders; our findings indicate otherwise. The small number of trainees who become research leaders are probably self‐motivated and should be supported. This leaves a substantial number of trainees who could contribute to worthwhile collaborative research enterprises (eg, participation in clinical trials and observational studies) but who are not currently being prepared or provided incentives to do so. In the United Kingdom, trainee research collaboratives have contributed to high quality research while developing trainee research skills since 2007.^32^, ^33^ Trainee research collaboratives are beginning to form across Australia and New Zealand,^34^ but the contributions of trainees rarely receive college recognition unless the trainees are the first authors on publications.

### Limitations

The overall response level for our survey was low. We took a pragmatic approach to recruitment to maximise sample size, which was largely determined by the individual colleges. Some colleges required an anonymous link for survey distribution, preventing tracking how each recruitment method contributed to survey participation. According to workforce data, about 43 500 current and past trainees were potentially eligible participants at the time of survey distribution (Supporting Information, table 7), but we cannot estimate how many were aware of the survey. We have more complete data from colleges that directly emailed their trainees, yielding response rates of 2.4% to 47%, although some people may have been recruited by other means (newsletters, snowballing etc.). Low survey response rates were typical at the time of survey distribution, the second year of the COVID‐19 pandemic, when many clinicians experienced survey fatigue and burnout.^35^, ^36^ Further, the small numbers of responses by college prevented analysis of differences between colleges.

Almost all participants (98%) reported how many projects they had completed, but only 45% responded to the subsequent question about project conduct. We cannot judge the representativeness of this sample, but our results are probably biased toward more positive experiences. First, fewer than 1% of Australian doctors identify as researchers,^37^ and fewer than 8% participate in research.^38^ In contrast, 33 of 61 respondents (54%) reported participating in research projects after completing training, and 44 (72%) had considered doing so. Second, the publication rate for uploaded project reports was higher (25 of 34, 74%) than the overall rate for survey participants (45 of 90), and for health and medical research more generally (50%),^39^, ^40^ possibly indicating better quality studies than for all trainees. Third, participants who subsequently participated in interviews described having more positive experiences than their peers.^19^

### Conclusions

Most of the current or recent trainees who participated in our survey were required to conduct research projects. Access to support, such as quality supervision, time, and research expertise, was inconsistent, as was satisfaction with the research experience. The quality of outputs, including publication rates and reporting quality, were also variable. Respondents noted some positive aspects, but voiced concerns about the quality of outputs, the time burden, and the lack of relevance for their careers. Our findings indicate that mandatory research projects are not an appropriate approach to research training for all specialist trainees and may have unintended consequences, including the contribution to low quality research findings of poorly planned and executed projects. While some trainees undertook high quality research, ensuring that every trainee undertakes a meaningful study is not feasible. Instead of leading research, trainees should be supported to hone research skills more relevant to their careers, as most aim to be evidence‐based clinicians and to engage in collaborative research. A research curriculum should be developed that is adaptable to individual career aspirations and training needs.

## Open access

Open access publishing facilitated by Griffith University, as part of the Wiley – Griffith University agreement via the Council of Australian University Librarians.

## Competing interests

Paulina Stehlik and Caitlin Brandenburg are members of the Queensland Training for Research Active Clinicians (QTRAC) working party. Caitlyn Withers is a plastic and reconstructive surgery trainee. Adrian Barnett and Paul Glasziou are members of the National Health and Medical Research Council research quality steering committee. Gerben Keijzers is a trainee research requirement adjudicator for the Australasian College of Emergency Medicine. Ian Scott gives lectures on research methods to trainees of the Royal Australasian College of Physicians and has been an examiner for the college. Emma Veysey is chair of the Australasian College of Dermatologists academic research committee. Mark Morgan is a clinical advisor and chair of Royal Australian College of General Practitioners expert committee for quality care, clinical advisor for Primary Sense (Australian general practice data extraction and analysis tool), and head of program for medical doctorates at Bond University. Hitesh Joshi is a member (casual) of the Royal Australian and New Zealand College of Psychiatrists committee for examinations. Kirsty Forrest is Dean of Medicine, Bond University, executive committee member and treasurer of Medical Deans of Australia and New Zealand, chair of the education and evaluation committee of the Australia and New Zealand College of Anaesthetists (ANZCA) and chair of professional practice research network at ANCZA. Thomas Campbell is on the curriculum committee for the Royal Australian and New Zealand College of Ophthalmologists.

## Data sharing

De‐identified participant data will be made available on request to the corresponding author.

## Supporting information


Supplementary methods and results
