# Supplementary material for: Mandatory research projects during medical specialist training in Australia and New Zealand: a survey of trainees’ experiences and reports
Source: Med J Aust. 2025 Feb 25;222(5):231–9. doi: 10.5694/mja2.52611 (PMC11910950; doi:10.5694/mja2.52611)
Supplement: Supplementary file 1 — Supplementary methods and results [file MJA2-222-231-s001.pdf]

The Medical Journal of Australia

Supporting Information

Supplementary methods and results

This appendix was part of the submitted manuscript and has been peer reviewed.
It is posted as supplied by the authors.

Appendix to: Stehlik P, Withers C, Bourke RC, et al. Mandatory research projects during medical specialist training in Australia and New Zealand: a survey of trainees' experiences and reports. *Med J Aust* 2025; doi: 10.5694/mja2.52611.

Supplementary methods

1. Additional information on survey procedure

Participants could access the survey via the Qualtrics platform. Incomplete responses were recorded. Respondents were also prevented from multiple submissions (setting on Qualtrics system: “This setting works by placing a cookie on their browser when they submit a response.”) The last email invitation was sent on 17 September 2021 and the survey remained open until 31 December 2021.

Participants could use the “back” button to change their responses and could return to surveys to complete them up to three months after closing their browser before their responses were recorded.

At the beginning of the survey, participants provided information used to generate a unique identifier based on their initials, day and month of birth, and gender, used to check for duplicate participants and to remove survey data at participant request. No participants requested that their data be removed. Potential duplicate responses were examined manually, and two response sets were identified as duplicates; the second response set was included in the analysis.

Participation was voluntary, and no incentives were offered to participants. Completion of the survey was assumed to reflect consent.

2. The survey

Participant eligibility check

Number	Question	Responses	Logic
0a	Are you currently completing your specialty training? OR Have completed your specialty training in the past 5 years (2016 onwards)?	Yes<br>No	No = end survey
0b	Did you complete or are you currently completing your speciality training through an Australian and/or New Zealand college?	Yes<br>No	No = end survey

The following questions were used to generate a unique identifier for participants so we could check for duplicates and to remove participants if they requested for their data to be removed.

Number	Question	Responses	Logic
0.1a	What are the **first 2** letters of your **first name**?	Free text	-
0.1b	What are the **last 2** letters of your **surname**?	Free text	-
0.1c	What are the **2 digits** of the day you were born on?<br>e.g. the 5th would be 05 and the 15th would be 15.	Number	-
0.1d	What are the **2 digits** of the **month** you were born in?<br>e.g. December would be 12.	Number	-
0.1e	Which gender do you identify with?	Male<br>Female<br>Non-binary<br>Prefer not to say	-

Main survey, part 1

We asked the following questions regarding participants **most recent** specialty training:

Number	Question	Responses	Logic
1	Have you finished your specialty training?	Yes<br>No	No = Skip Q2
2	What year did you complete your most recent specialty training? (e.g. 2019)	Number	
3	Which college did you complete/ are completing your specialty training with?<br><br>If you completed more than one specialty training at a time (e.g. dual RACP/RCPA training) please choose the most recent one.<br><br>If no sub-specialty, put "-".	Drill down list with all Australian and New Zealand specialties and subspecialties	Other = complete Q3.1

Number	Question	Responses	Logic
	If your most recent specialty training is not on the list, please select College as "OTHER" and Sub specialty as " - "		
3.1	Please specify which college and subspecialty you completed/ are completing your most recent specialty your training with:	Free text	-
4	Which country and state did you complete/ are completing **most** of your most recent specialty training in?<br>If you completed your most recent training in New Zealand or OTHER, please put "-" as the state.	Drill down options with All Australian states and New Zealand	
5	In which geographic area did you complete/ are completing **most** of your most recent specialty training?	Urban<br>Regional<br>Rural<br>Remote	-
6	Did you complete one or more scholarly project(s) as part of your specialty training?<br><br>For the purposes of this survey, a **scholarly project** is any project type work that was mandated by the college as part of your specialty training.<br><br>This includes any of the following: primary research (e.g. randomised trials, cohort studies, case controls, etc) secondary research (e.g. systematic review, scoping review, literature review, narrative review), case reports or case series, quality improvement, large audits, and qualitative research.	Yes<br>In progress<br>I plan to<br>No	If Yes, go to next question (Q7).<br><br>If No, go to Q11.<br><br>Otherwise go to Q12
7	How important did you feel conducting a scholarly project was to your clinical career development?	Very important<br>Moderately important<br>Slightly important<br>Not at all important	Only see if Q6 = Yes
8	How many scholarly projects were you required to undertake as part of your college mandated requirements? (Please answer in numerals.)	Number	Used to determine how many times participants would need to complete the next section (Q9)

Individual projects

In this section participants were asked the following questions for each of their projects. If they said they had to complete two projects, they would see the following questions twice.

Participants only saw this section if they responded YES to Q6; otherwise this section was skipped.

Number	Question	Responses	Logic
9a	**NEW PROJECT UPLOAD**<br>Please upload a copy of your submission to the college (e.g.: thesis, manuscript, conference abstract, case report, etc).<br><br>If you were required to submit a protocol *and* final report for the same project, please submit the final report ONLY.<br><br>We will be characterising your upload, including study design and analysis used. We will not use your upload for anything other than for the purposes of this study. Your submission will be deidentified prior to any analysis and will be held securely on Bond servers. We will NOT make any aspect of your project public.<br><br>Note: Please ensure that the name of your file is less than 10 characters long. If you would like to change the file you have uploaded, simply click on the light-grey upload area and choose the file you would like to upload.<br><br>Please note that uploading your submission is optional - you are unable to find your submission or to not wish to upload a copy of your submission, please go to the next question.	File upload	OPTIONAL – participants did not have to upload a file.
9b	Which best describes the circumstances in which you generated your **research question**?	On my own<br>It was a component of an ongoing project - e.g. part of a grant, one of the department priority area projects, etc<br>A result of a clinical discussion- e.g. recommended by my supervisor after a clinical meeting.<br>Other	-
9c	Before starting your project, did you search for a systematic review or other type of review (e.g. scoping review) that	Yes<br>No	-

Number	Question	Responses	Logic
	answered your question prior to starting your research?		
9d	Which best describes the circumstances in which you generated your **study design**?	On my own without or with minimal input from others.<br>On my own but with significant input from others.<br>The protocol was part of an existing project	-
9e	Before starting data collection for this project, did you develop research protocol?	Yes - I developed one myself<br>Yes - there one already developed<br>No	If Yes - I developed one myself go to Q9f
9f	Was the protocol registered in a publicly available place? (Tick all that apply)	Yes - Published in a journal<br>Yes - in a registry (e.g. PROSPERO, ClinicalTrials.gov, OSF, etc):<br>Yes - Other. Please state where:<br>No	
9g	Were consumers involved in the design of your research?<br><br>Note, For the purposes of this question, 'consumers' are people who have lived experience of the health issue or topic under investigation.	Yes<br>No	If no, skip to 9j
9h	Please state which part of the research process the consumers were involved in (tick all that apply).	Developing the research question<br>Protocol design<br>Conduct of research<br>Dissemination of research<br>Future work including implementation of research findings and/or developing future research questions	
9i	What level of involvement did the consumer have?	Consultation<br>Co-investigator/collaborator<br>Lead	
9j	Did your research team consist of members outside of your own profession? (Tick all that apply).	Yes - Medical professional(s) from a different specialty. Please specify:<br>Yes - Allied Health Professional(s). Please specify:<br>Yes - Nursing staff.	

Number	Question	Responses	Logic
		Yes -Statistician(s).<br>Yes - Health economist(s).<br>Yes - Librarian(s)/ Information Specialist(s)<br>Yes - Data scientist(s)<br>Yes - Other. Please specify:<br>No	
9k	Were the results of your study presented to the department where are doing or did your clinical training?	Yes<br>No	
9l	Do you or your colleagues believe that the results of this study may be useful in practice?	Yes<br>No	
9m	How confident are you in using the findings of your study in clinical practice?	Very confident<br>Somewhat confident<br>Not at all confident	
9n	Is a manuscript containing the results publicly available?	Yes - Published in a journal by the end of your training<br>Yes - Subsequently published in a journal<br>Yes - Pre-print available<br>No - It is unpublished	
9o	Please provide the citation of your work in the Vancouver format as per the picture below, even if unpublished, OR a DOI link.<br>Vancouver citation example:<br>Stehlik P., Noble C., Brandenburg C., Fawzy P., Narouz I., Henry D., Glasziou P. How do trainee doctors learn about research? Content analysis of Australian specialist colleges' intended research curricula. BMJ Open. 2020;10:e034962. doi: 10.1136/bmjopen-2019-034962<br><br>DOI example:<br>http://dx.doi.org/10.1136/bmjopen-2019-034962; OR<br>10.1136/bmjopen-2019-034962	Free text	If 9n = No, this was skipped
9p	Which author position did you have for this publication?	First<br>Second<br>Last<br>Other	If 9n = No, this was skipped
9q	We are interested in when you completed your project.<br>What was the approximate percentage (%) of time spent conducting your project? Please round to the nearest 5% (Note: the total % should add up to 100%):	Number scale used to put in under the following headings:<br>During scheduled service/clinical work time?	

Number	Question	Responses	Logic
		During protected time - e.g. grant funded time, time allocated for research, etc?<br>In your own time?	
9r	Please indicate the extent to which you agree or disagree with the following statement.<br>My supervisor provided me with adequate research support while conducting your scholarly project.	Strongly agree<br>Agree<br>Neither agree nor disagree<br>Disagree<br>Strongly disagree<br>I did not have a supervisor	
9s	Please indicate if you had adequate access to any of the following types of individuals while completing your scholarly project (tick if yes to any that apply):	Statistician(s)<br>Health economist(s)<br>Librarian(s)<br>Consumer or patient advocate(s)<br>Experts in research design or measurement<br>Experts in practice change strategies or practice improvement<br>Individuals with sufficient breadth and depth of clinical expertise<br>None of the above	

About the overall experience

This section was asked once (unlike the previous section which was per project) to participants who responded Yes to Q6.

Participants were about their **overall** experience in undertaking a scholarly project during their most recent specialty training and extent to which they agreed or disagreed with each of statement.

Number	Question	Responses	Logic
10a	I had the necessary knowledge and skills to complete my scholarly project(s).	Strongly agree<br>Agree<br>Neither agree nor disagree<br>Disagree<br>Strongly disagree	
10b	I had access to a good research-related seminar(s) or training program.	Strongly agree<br>Agree<br>Neither agree nor disagree<br>Disagree<br>Strongly disagree	
10c	Completing a scholarly project(s) during my specialty training gave me a better understanding of how to read and interpret other people's research.	Strongly agree<br>Agree<br>Neither agree nor disagree<br>Disagree<br>Strongly disagree	

Number	Question	Responses	Logic
10d	Overall, I was satisfied with the quality of my research experience during my specialty training	Strongly agree<br>Agree<br>Neither agree nor disagree<br>Disagree<br>Strongly disagree	

Reasons for not completing a project

The following section was to understand what the reasons might be for participants to not be required to complete a scholarly project – e.g. recognition of prior learning, alternative pathways, or not a requirement.

Only participants who responded No to Q6 saw this section

Number	Question	Responses	Logic
11	What was the reason you did not complete a scholarly project?	It was not required<br>I had recognition of prior learning<br>I completed a PhD instead<br>I completed a research Masters instead<br>I completed approved coursework instead<br>Other:	Only seen if Q6 = No

Other questions

All participants saw these questions unless indicated otherwise in the logic column.

Number	Question	Responses	Logic
12	How much do you support or oppose the requirement to complete a scholarly project during specialty training?	Strongly support<br>Moderately support<br>Neither support nor oppose<br>Moderately oppose<br>Strongly oppose	-
13	Please provide the reasons behind your response to the question above.	Free text	-
14	Since gaining your most recent fellowship, have you considered initiating a new research project?	Yes<br>No	If Q1 = Yes
14b	Please explain why or why not.	Free text	If Q1 = Yes
14c	Since gaining your most recent fellowship, have you participated in any research projects as an investigator?	Yes<br>No	If Q1 = Yes
14d	Please explain why or why not.	Free text	If Q1 = Yes
15	Do you have any additional/ final comments?	Free text	-
16	Do you wish to answer additional questions regarding your research experience during your specialty training? This will take approximately 30 mins to complete.	Yes<br>No	If Q6 = Yes

3. Quality assessment of research outputs

We assessed each uploaded project in two rounds of data extraction. During the first round, we categorised the submission type, research question type, study design, and whether the upload was an audit. We assessed whether the authors asked a clear research question, provided a study rationale, adequately considered the published literature, or provided a sample size calculation (if relevant). During data extraction, we noticed that many projects did not explicitly label, or mislabelled, the study design; we therefore added this as an extra variable during data extraction. For published manuscripts, we checked whether the journal stated they used a peer-review process, and whether the journal was listed on the Predatory Journal list (https://predatoryreports.org/the-list).

During the second round of data extraction, we assessed the quality of reporting and design of each upload. We used EQUATOR-network (https://www.equator-network.org/reporting-guidelines) reporting guidelines to assess the quality of reporting of individual studies (Table 1). We originally planned to use risk of bias tools recommended by the Cochrane Collaboration to assess study quality, but the variety of study questions and designs would have made it difficult to interpret results from several different tools and we therefore modified them as described in Table 1.

Given that some uploads mislabelled their study design or did not provide a study design we used the following rules to decide on the quality assessment tool. For studies that incorrectly labelled their study, we assessed the quality of reporting based on the study design they assigned themselves and the design quality assessment on the actual study design. For those that did not provide a study design label, we assigned the study design based on information in their methods section and used the relevant reporting and design quality tools. We excluded studies from quality assessment if a reporting guideline or critical appraisal instrument was not available.

All data extraction was done independently by two authors. Discrepancies were resolved by discussion, or by discussion with a third author with the relevant skill set (AB was used for all statistical resolutions and DH and PG were used for all methodological/design resolutions).

Table 1. Reporting and design quality assessment tools

Tool	Study designs	Number of projects that underwent assessment
**Reporting quality**		
AGREE(1)	Guidelines	1
ARRIVE(2)	Animal studies	1
CARE(3)	Case reports, case series	4
CONSORT Cross-over(4)	Cross over randomised trial	1
PRISMA (5)	Systematic reviews	6
PRISMA-ScR (6)	Scoping reviews	2
SRQR (7)	Qualitative	1
STARD (8)	Diagnostic test accuracy	2
STROBE (9)	Cohort, case–control, cross-sectional	10
**Design quality**		
MMAT(10)	Animal studies*, case reports, case series, cross over randomised trial, qualitative, cohort, case–control, cross sectional	16
Modified AMSTAR-2 (11)†	Systematic review, scoping review	8
QUADAS-2 (12)‡	Diagnostic test accuracy	2
MiChe (13)	Guidelines	1

* One laboratory animal study was a randomised trial and could not be evaluated with the MMAT quantitative randomized controlled trial tool.

† To allow for evaluation of non-interventional studies and scoping reviews, the AMSTAR signalling questions were modified by three team members, one of whom was an author on the original AMSTAR tool (David A Henry), a statistician (Adrian Barnett), and a systematic review expert (Alexandra Bannach-Brown).

‡ Risk of bias elements only.

Supplementary results

Table 2. Additional demographic data*

	Participants	
**Answer**	**Number**	**Proportion**
**Participants who provided any survey data**		
**Specialty training college**	**371/371**	**100%**
Australasian College of Dermatologists	3	1%
Australasian College for Emergency Medicine	26	7%
Australian College of Rural and Remote Medicine	2	1%
Australasian College of Sport and Exercise Physicians	5	1%
Australian and New Zealand College Of Anaesthetists	86	23%
College of Intensive Care Medicine	22	6%
Royal Australasian College of Dental Surgeon	1	0
Royal Australian College of General Practitioners	27	7%
Royal Australasian College of Medical Administrators	0	0
Royal Australasian College of Physicians	102	27%
Royal Australasian College of Surgeons	23	6%
Royal Australian and New Zealand College of Ophthalmologists	9	2%
Royal Australian and New Zealand College of Obstetricians and Gynaecologists	11	3%
Royal Australian and New Zealand College of Psychiatrists	36	10%
Royal Australian and New Zealand College of Radiologists	5	1%
Royal College of Pathologists of Australasia	9	2%
Other/ Prefer not to say	4	2%
**Year training was completed**	**133/133**	**100%**
2015[†]	2	2%
2016	11	8%
2017	22	17%
2018	21	16%
2019	22	17%
2020	55	41%
**Which country and state did you complete/are completing most of your most recent specialty training in?**	**365/371**	**98%**
Australia: Australian Capital Territory	3	1%
Australia: New South Wales	75	20%
Australia: Northern Territory	2	1%
Australia: Queensland	102	27%
Australia: South Australia	16	4%
Australia: Tasmania	4	1%
Australia: Victoria	74	20%
Australia: Western Australia	38	10%
New Zealand: -	48	13%
Other: -	3	1%

	Participants	
**Answer**	**Number**	**Proportion**
**Participants who provided project information**		
**Specialty training college**	**79/177‡**	**45%**
Australasian College of Dermatologists	0	0
Australasian College for Emergency Medicine	2	3%
Australian College of Rural and Remote Medicine	0	0
Australasian College of Sport and Exercise Physicians	2	3%
Australian and New Zealand College of Anaesthetists	18	23%
College of Intensive Care Medicine	11	14%
Royal Australasian College of Dental Surgeon	1	1%
Royal Australian College of General Practitioners	3	4%
Royal Australasian College of Medical Administrators	0	0
Royal Australasian College of Physicians	16	20%
Royal Australasian College of Surgeons	8	10%
Royal Australian and New Zealand College of Ophthalmologists	0	0
Royal Australian and New Zealand College of Obstetricians and Gynaecologists	1	1%
Royal Australian and New Zealand College of Psychiatrists	11	14%
Royal Australian and New Zealand College of Radiologists	2	3%
Royal College of Pathologists of Australasia	4	5%
Other	0	0

* Survey logic meant that those eligible to respond to each question changed throughout the survey; proportions of those eligible who answered are provided. Total answers are used as the denominator for each question.

† These two participants had passed the eligibility test, so it appears that the date entered may have been an error. One of these participants contributed project data for a single project, but provided no uploads for analysis.

‡ 177 participants said that they had completed a project

Table 3. Characteristics of the research projects and research teams in the projects undertaken by the participants in our 2021 medical specialty trainee mandatory research project survey*

	Projects	
**Answer**	**Number**	**Proportion**
**Generation of study question**	**92/267**	**34%**
On my own	38	41%
It was a component of an ongoing project - e.g. part of a grant, one of the department priority area projects, etc	14	15%
A result of a clinical discussion- e.g. recommended by my supervisor after a clinical meeting.	35	38%
Other	5	5%
**Searched for a review before to starting project**	**92/267**	**34%**
Yes	68	74%
No	24	26%
**Protocol developed before data collection**	**92/267**	**34%**
Yes - I developed one myself	60	65%
Yes - there one already developed	9	10%
No	23	25%
**Protocol registered in a publicly available place†‡**	**60/60**	**100%**
Yes - Published in a journal	11	18%
Yes - in a registry	7	11%
Yes - Other. Please state where:	3	5%
No	40	66%
**Consumers involved in research**	**90/267**	**34%**
Yes	7	8%
No	83	92%
**Which part of the research process the consumers were involved in?‡**	**7/7**	**100%**
Developing the research question	0	0%
Protocol design	3	42%
Conduct of research	6	85%
Dissemination of research	2	28%
Future work including implementation of research findings and/or developing future research questions	3	42%
**Which part of the research process the consumers were involved in?**	**7/7**	**100%**
Consultation	7	100%
Co-investigator/collaborator	0	0%
Lead	0	0%
**Research team consisted of members outside of trainee's own profession‡**	**90/267**	**34%**
Yes - Medical professional(s) from a different specialty.	8	8%
Yes - Allied Health Professional(s)	10	11%
Yes - Nursing staff.	10	11%
Yes -Statistician(s).	21	23%
Yes - Health economist(s).	1	1%
Yes - Librarian(s)/ Information Specialist(s).	8	8%
Yes - Data scientist(s).	5	5%
Yes - Other.	9	10%

	Projects	
**Answer**	**Number**	**Proportion**
No	39	43%
**Adequate access to relevant expertise to while completing scholarly project(s)‡**	**85/267**	**32%**
Statistician(s)	7	8%
Health economist(s)	0	0%
Librarian(s)	22	25%
Consumer or patient advocate(s)	2	2%
Experts in research design or measurement	17	20%
Experts in practice change strategies or practice improvement	8	9%
Individuals with sufficient breadth and depth of clinical expertise	45	52%
No	17	20%
**Trainee or colleagues believe that the results are useful in practice**	89/267	33%
Yes	78	88%
No	11	12%
**Results presented to department**	**90/267**	**34%**
Yes	68	76%
No	22	24%
**Results are publicly available‡**	**90/267**	**34%**
Yes - Published in a journal by the end of your training	33	37%
Yes - Subsequently published in a journal	12	13%
Yes - Pre-print available	1	1%
No	44	49%
**Which author position did you have for this publication?**	42/45	93%
First	37	88%
Second	4	10%
Last	1	2%
Other	0	0
**How confident are you in using the findings of your study in clinical practice?**	**90/267**	**34%**
Very confident	39	43%
Somewhat confident	42	47%
Not at all confident	9	10%

	Participants	
**Answer**	**Number**	**Proportion**
How important did you feel conducting a scholarly project was to your clinical career development?	174/177	98%
Very important	30	17%
Moderately important	57	33%
Slightly important	56	32%
Not at all important	31	18%
**Has considered initiating new research since completing training**	**61/133**	**46%**
Yes	44	72%
No	17	28%

**Has participated in research projects since completing training**	**61/133**	**46%**
Yes	33	54%
No	28	46%

* Survey logic meant that those eligible to respond to each question changed throughout the survey; proportions of those eligible who answered are provided. Total answers are used as the denominator for each question.

† Survey logic was incorrectly implemented, so that only those who had developed a protocol themselves were asked this question rather than all those who responded yes to developing a protocol.

‡ Project numbers add to more than those that answered as each project could have answered “yes” to more than one category.

Table 4. Time allocation to research projects undertaken by the participants in our 2021 medical specialty trainee mandatory research project survey

			Median proportion (interquartile range)		
**College**	**Participants**	**Projects**	**Clinical time**	**Protected time**	**Own time**
Australasian College for Emergency Medicine	2	2	5% (2–7%)	5% (2–7%)	90% (90–90%)
Australasian College of Sport and Exercise Physicians	2	2	28.5% (24–32%)	15.5% (7–23%)	56% (44–68%)
Australian and New Zealand College Of Anaesthetists	15	20*	7% (3–16%)	0 (0–1%)	90% (71–95%)
College of Intensive Care Medicine	10	10	4.5% (0–5%)	0 (0–3%)	92.5% (89–98%)
Royal Australasian College of Dental Surgeons	1	2†	25%	0%	75%
Royal Australian College of General Practitioners	3	3	5% (2–6%)	85% (80–89%)	10% (8–14%)
Royal Australasian College of Physicians	11	14‡	8% (0–10%)	6% (0–20%)	81% (71–94%)
Royal Australasian College of Surgeons	8	8	5% (4%–16%)	2.5% (0–27%)	86% (53–95%)
Royal Australian and New Zealand College of Obstetricians and Gynaecologists	1	1	0 (0–0%)	0 (0–0%)	100% (100–100%)
Royal Australian and New Zealand College of Psychiatrists	11	13‡	10% (0–11%)	19% (0–25%)	75% (52–89%)
Royal Australian and New Zealand College of Radiologists	2	3†	0% (0–1%)	0 (0–1%)	100% (98–100%)
Royal College of Pathologists of Australasia	4	7§	90% (7–100%)	0 (0–0%)	10% (0–92%)
Overall	72	85	5% (0–16%)	0 (0–11%)	89 (66–95%)

We asked participants to estimate the proportion of time they spent on their scholarly projects during scheduled service/clinical time, protected time, and during their own time. We received responses for 85 of 267 projects. Red highlights indicate largest median proportion for the row. The RACGP trainee program offers an academic post that provides funding for protected research time.

* Five participants provided information on two projects each.

† One participant provided information on two projects.

‡ Two participants provided information on two projects each.

§ One participant provided information on four projects.

Table 5. Research report manuscripts uploaded by the participants in our 2021 medical specialty trainee mandatory research project survey

Answer	Manuscripts
**All uploaded report manuscripts**	34
**College**	
Royal Australian and New Zealand College of Psychiatrists	8
College of Intensive Care Medicine	7
Australian and New Zealand College of Anaesthetists*	7
Royal College of Pathologists of Australasia†	4
Royal Australasian College of Surgeons	3
Royal Australasian College of Physicians	2
Royal Australian College of General Practitioners	1
Royal Australian and New Zealand College of Obstetricians and Gynaecologists	1
Australasian College for Emergency Medicine	1
**What type of submission was provided?**	
Published manuscript‡	25
Manuscript/report	8
Poster	1
**Was this an audit or QI project?**	
No	32
Yes - ad-hoc§	2
**Did the authors provide a sound argument for the rationale to do the study and/or that the results of the study provide meaningful information?**	
Yes	31
No	3
**Was there an adequate consideration of the published literature on the topic, including previous systematic reviews?**	
Yes	23
No	10
Not applicable¶	1
**Was there a clear, well-structured and answerable research question? (e.g. PICO-T).**	
Yes	28
No	4
Partial	2
**What type of question did the researchers ask?**	
Intervention	11
Prevalence	9
Other	5
Diagnostic test accuracy	3
Risk	2
Prognosis	2
Phenomenology	1
Rate	1
**What study design did the researchers use?**	
Cross sectional	10

Answer	Manuscripts
Review - systematic review	6
Case series	4
Review - scoping review	2
Mathematical modelling	2
Cohort with control	2
Randomised control trial - cross over	1
Qualitative - descriptive	1
In-vitro	1
Cohort without control	1
Review - Narrative or literature review	1
Clinical practice guideline	1
Case control	1
Preclinical - animal study	1
**Did the method stated match what was described?**	
Yes	20
None given	13
No	1
**Was there a sample size calculation or was the power of the study to provide a meaningful result discussed?**	
Not applicable	21
No	11
Yes	2

* Two individuals each uploaded two articles.

† All four articles were from the same individual.

‡ One published in a potentially predatory journal.

§ “It was not made clear in the manuscript that the audit or quality improvement project was part of a pre-specified local or state/national project.

¶ This upload was a poster that did not provide a comprehensive literature overview.

Figure 1. Quality of reporting in 28 research report manuscripts or articles uploaded by the participants in our 2021 medical specialty trainee mandatory research project survey*

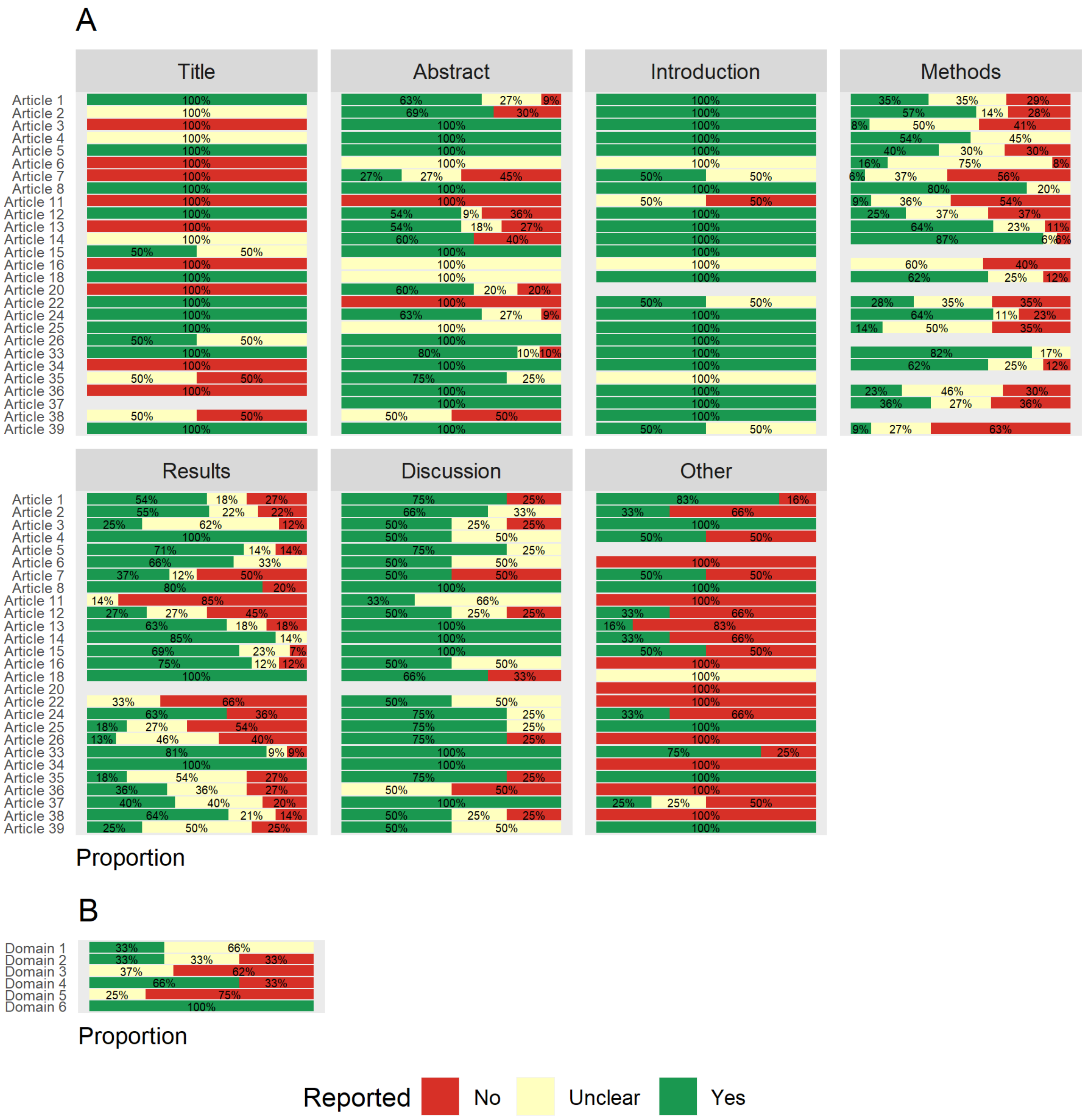

* Panel A includes assessments for 27 of the 28 evaluated manuscripts; panel B shows the assessment of a clinical guideline using the AGREE tool because its structure was different to those of the other. Domain 1: Scope And Purpose; Domain 2: Stakeholder Involvement; Domain 3: Rigour Of Development; Domain 4: Clarity Of Presentation; Domain 5: Applicability; Domain 6: Editorial Independence. In some cases, specific aspects could not be assessed because of the manuscript type: poster (article 20); CARE(3) reporting guideline for case series and case reports do not require methods sections (articles 15, 26, 35, 38); the ARRIVE (3) reporting guideline for animal studies merges title and abstract (article 37). The data underlying this figure are included in table 6.

Table 6. Quality of reporting in 28 research report manuscripts or articles uploaded by the participants in our 2021 medical specialty trainee mandatory research project survey*

		Number of elements reported			Proportion of elements reported		
		No	Unclear/Partial	Yes	No	Unclear/Partial	Yes
Article	Section	Value	Value	Value	Prop.	Prop.	Prop.
**Article 1**	Title	0/1	0/1	1/1	0%	0%	100%
**Article 1**	Abstract	1/11	3/11	7/11	9%	27%	63%
**Article 1**	Introduction	0/2	0/2	2/2	0%	0%	100%
**Article 1**	Methods	5/17	6/17	6/17	29%	35%	35%
**Article 1**	Results	3/11	2/11	6/11	27%	18%	54%
**Article 1**	Discussion	1/4	0/4	3/4	25%	0%	75%
**Article 1**	Other	1/6	0/6	5/6	16%	0%	83%
**Article 2**	Title	0/1	1/1	0/1	0%	100%	0%
**Article 2**	Abstract	4/13	0/13	9/13	30%	0%	69%
**Article 2**	Introduction	0/2	0/2	2/2	0%	0%	100%
**Article 2**	Methods	4/14	2/14	8/14	28%	14%	57%
**Article 2**	Results	2/9	2/9	5/9	22%	22%	55%
**Article 2**	Discussion	0/3	1/3	2/3	0%	33%	66%
**Article 2**	Other	2/3	0/3	1/3	66%	0%	33%
**Article 3**	Title	1/1	0/1	0/1	100%	0%	0%
**Article 3**	Abstract	0/1	0/1	1/1	0%	0%	100%
**Article 3**	Introduction	0/2	0/2	2/2	0%	0%	100%
**Article 3**	Methods	5/12	6/12	1/12	41%	50%	8%
**Article 3**	Results	1/8	5/8	2/8	12%	62%	25%
**Article 3**	Discussion	1/4	1/4	2/4	25%	25%	50%
**Article 3**	Other	0/1	0/1	1/1	0%	0%	100%
**Article 4**	Title	0/1	1/1	0/1	0%	100%	0%
**Article 4**	Abstract	0/1	0/1	1/1	0%	0%	100%
**Article 4**	Introduction	0/2	0/2	2/2	0%	0%	100%
**Article 4**	Methods	0/11	5/11	6/11	0%	45%	54%
**Article 4**	Results	0/2	0/2	2/2	0%	0%	100%
**Article 4**	Discussion	0/2	1/2	1/2	0%	50%	50%
**Article 4**	Other	1/2	0/2	1/2	50%	0%	50%
**Article 5**	Title	0/1	0/1	1/1	0%	0%	100%
**Article 5**	Abstract	0/1	0/1	1/1	0%	0%	100%
**Article 5**	Introduction	0/2	0/2	2/2	0%	0%	100%
**Article 5**	Methods	3/10	3/10	4/10	30%	30%	40%
**Article 5**	Results	1/7	1/7	5/7	14%	14%	71%
**Article 5**	Discussion	0/4	1/4	3/4	0%	25%	75%
**Article 5**	Other	NA	NA	NA	NA	NA	NA
**Article 6**	Title	1/1	0/1	0/1	100%	0%	0%
**Article 6**	Abstract	0/1	1/1	0/1	0%	100%	0%
**Article 6**	Introduction	0/2	2/2	0/2	0%	100%	0%
**Article 6**	Methods	1/12	9/12	2/12	8%	75%	16%

		Number of elements reported			Proportion of elements reported		
		No	Unclear/Partial	Yes	No	Unclear/Partial	Yes
**Article 6**	Results	0/9	3/9	6/9	0%	33%	66%
**Article 6**	Discussion	0/4	2/4	2/4	0%	50%	50%
**Article 6**	Other	1/1	0/1	0/1	100%	0%	0%
**Article 7**	Title	1/1	0/1	0/1	100%	0%	0%
**Article 7**	Abstract	5/11	3/11	3/11	45%	27%	27%
**Article 7**	Introduction	0/2	1/2	1/2	0%	50%	50%
**Article 7**	Methods	9/16	6/16	1/16	56%	37%	6%
**Article 7**	Results	4/8	1/8	3/8	50%	12%	37%
**Article 7**	Discussion	2/4	0/4	2/4	50%	0%	50%
**Article 7**	Other	3/6	0/6	3/6	50%	0%	50%
**Article 8**	Title	0/1	0/1	1/1	0%	0%	100%
**Article 8**	Abstract	0/1	0/1	1/1	0%	0%	100%
**Article 8**	Introduction	0/2	0/2	2/2	0%	0%	100%
**Article 8**	Methods	0/10	2/10	8/10	0%	20%	80%
**Article 8**	Results	1/5	0/5	4/5	20%	0%	80%
**Article 8**	Discussion	0/4	0/4	4/4	0%	0%	100%
**Article 8**	Other	0/1	0/1	1/1	0%	0%	100%
**Article 11**	Title	1/1	0/1	0/1	100%	0%	0%
**Article 11**	Abstract	1/1	0/1	0/1	100%	0%	0%
**Article 11**	Introduction	1/2	1/2	0/2	50%	50%	0%
**Article 11**	Methods	6/11	4/11	1/11	54%	36%	9%
**Article 11**	Results	6/7	1/7	0/7	85%	14%	0%
**Article 11**	Discussion	0/3	2/3	1/3	0%	66%	33%
**Article 11**	Other	1/1	0/1	0/1	100%	0%	0%
**Article 12**	Title	0/1	0/1	1/1	0%	0%	100%
**Article 12**	Abstract	4/11	1/11	6/11	36%	9%	54%
**Article 12**	Introduction	0/2	0/2	2/2	0%	0%	100%
**Article 12**	Methods	6/16	6/16	4/16	37%	37%	25%
**Article 12**	Results	5/11	3/11	3/11	45%	27%	27%
**Article 12**	Discussion	1/4	1/4	2/4	25%	25%	50%
**Article 12**	Other	4/6	0/6	2/6	66%	0%	33%
**Article 13**	Title	1/1	0/1	0/1	100%	0%	0%
**Article 13**	Abstract	3/11	2/11	6/11	27%	18%	54%
**Article 13**	Introduction	0/2	0/2	2/2	0%	0%	100%
**Article 13**	Methods	2/17	4/17	11/17	11%	23%	64%
**Article 13**	Results	2/11	2/11	7/11	18%	18%	63%
**Article 13**	Discussion	0/4	0/4	4/4	0%	0%	100%
**Article 13**	Other	5/6	0/6	1/6	83%	0%	16%
**Article 14**	Title	0/1	1/1	0/1	0%	100%	0%
**Article 14**	Abstract	4/10	0/10	6/10	40%	0%	60%
**Article 14**	Introduction	0/2	0/2	2/2	0%	0%	100%
**Article 14**	Methods	1/16	1/16	14/16	6%	6%	87%

		Number of elements reported			Proportion of elements reported		
		No	Unclear/Partial	Yes	No	Unclear/Partial	Yes
**Article 14**	Results	0/7	1/7	6/7	0%	14%	85%
**Article 14**	Discussion	0/2	0/2	2/2	0%	0%	100%
**Article 14**	Other	2/3	0/3	1/3	66%	0%	33%
**Article 15**	Title	0/2	1/2	1/2	0%	50%	50%
**Article 15**	Abstract	0/4	0/4	4/4	0%	0%	100%
**Article 15**	Introduction	0/1	0/1	1/1	0%	0%	100%
**Article 15**	Methods	NA	NA	NA	NA	NA	NA
**Article 15**	Results	1/13	3/13	9/13	7%	23%	69%
**Article 15**	Discussion	0/4	0/4	4/4	0%	0%	100%
**Article 15**	Other	1/2	0/2	1/2	50%	0%	50%
**Article 16**	Title	1/1	0/1	0/1	100%	0%	0%
**Article 16**	Abstract	0/1	1/1	0/1	0%	100%	0%
**Article 16**	Introduction	0/2	2/2	0/2	0%	100%	0%
**Article 16**	Methods	4/10	6/10	0/10	40%	60%	0%
**Article 16**	Results	1/8	1/8	6/8	12%	12%	75%
**Article 16**	Discussion	0/4	2/4	2/4	0%	50%	50%
**Article 16**	Other	1/1	0/1	0/1	100%	0%	0%
**Article 18**	Title	0/1	0/1	1/1	0%	0%	100%
**Article 18**	Abstract	0/1	1/1	0/1	0%	100%	0%
**Article 18**	Introduction	0/2	0/2	2/2	0%	0%	100%
**Article 18**	Methods	1/8	2/8	5/8	12%	25%	62%
**Article 18**	Results	0/4	0/4	4/4	0%	0%	100%
**Article 18**	Discussion	1/3	0/3	2/3	33%	0%	66%
**Article 18**	Other	0/1	1/1	0/1	0%	100%	0%
**Article 20**	Title	1/1	0/1	0/1	100%	0%	0%
**Article 20**	Abstract	2/10	2/10	6/10	20%	20%	60%
**Article 20**	Introduction	NA	NA	NA	NA	NA	NA
**Article 20**	Methods	NA	NA	NA	NA	NA	NA
**Article 20**	Results	NA	NA	NA	NA	NA	NA
**Article 20**	Discussion	NA	NA	NA	NA	NA	NA
**Article 20**	Other	2/2	0/2	0/2	100%	0%	0%
**Article 22**	Title	0/1	0/1	1/1	0%	0%	100%
**Article 22**	Abstract	1/1	0/1	0/1	100%	0%	0%
**Article 22**	Introduction	0/2	1/2	1/2	0%	50%	50%
**Article 22**	Methods	5/14	5/14	4/14	35%	35%	28%
**Article 22**	Results	6/9	3/9	0/9	66%	33%	0%
**Article 22**	Discussion	0/4	2/4	2/4	0%	50%	50%
**Article 22**	Other	1/1	0/1	0/1	100%	0%	0%
**Article 24**	Title	0/1	0/1	1/1	0%	0%	100%
**Article 24**	Abstract	1/11	3/11	7/11	9%	27%	63%
**Article 24**	Introduction	0/2	0/2	2/2	0%	0%	100%
**Article 24**	Methods	4/17	2/17	11/17	23%	11%	64%

		Number of elements reported			Proportion of elements reported		
		No	Unclear/Partial	Yes	No	Unclear/Partial	Yes
**Article 24**	Results	4/11	0/11	7/11	36%	0%	63%
**Article 24**	Discussion	0/4	1/4	3/4	0%	25%	75%
**Article 24**	Other	4/6	0/6	2/6	66%	0%	33%
**Article 25**	Title	0/1	0/1	1/1	0%	0%	100%
**Article 25**	Abstract	0/1	1/1	0/1	0%	100%	0%
**Article 25**	Introduction	0/2	0/2	2/2	0%	0%	100%
**Article 25**	Methods	5/14	7/14	2/14	35%	50%	14%
**Article 25**	Results	6/11	3/11	2/11	54%	27%	18%
**Article 25**	Discussion	0/4	1/4	3/4	0%	25%	75%
**Article 25**	Other	0/1	0/1	1/1	0%	0%	100%
**Article 26**	Title	0/2	1/2	1/2	0%	50%	50%
**Article 26**	Abstract	0/4	0/4	4/4	0%	0%	100%
**Article 26**	Introduction	0/1	0/1	1/1	0%	0%	100%
**Article 26**	Methods	NA	NA	NA	NA	NA	NA
**Article 26**	Results	6/15	7/15	2/15	40%	46%	13%
**Article 26**	Discussion	1/4	0/4	3/4	25%	0%	75%
**Article 26**	Other	1/1	0/1	0/1	100%	0%	0%
**Article 33**	Title	0/1	0/1	1/1	0%	0%	100%
**Article 33**	Abstract	1/10	1/10	8/10	10%	10%	80%
**Article 33**	Introduction	0/2	0/2	2/2	0%	0%	100%
**Article 33**	Methods	0/17	3/17	14/17	0%	17%	82%
**Article 33**	Results	1/11	1/11	9/11	9%	9%	81%
**Article 33**	Discussion	0/4	0/4	4/4	0%	0%	100%
**Article 33**	Other	1/4	0/4	3/4	25%	0%	75%
**Article 34**	Title	1/1	0/1	0/1	100%	0%	0%
**Article 34**	Abstract	0/1	0/1	1/1	0%	0%	100%
**Article 34**	Introduction	0/2	0/2	2/2	0%	0%	100%
**Article 34**	Methods	1/8	2/8	5/8	12%	25%	62%
**Article 34**	Results	0/4	0/4	4/4	0%	0%	100%
**Article 34**	Discussion	0/3	0/3	3/3	0%	0%	100%
**Article 34**	Other	1/1	0/1	0/1	100%	0%	0%
**Article 35**	Title	1/2	1/2	0/2	50%	50%	0%
**Article 35**	Abstract	0/4	1/4	3/4	0%	25%	75%
**Article 35**	Introduction	0/1	1/1	0/1	0%	100%	0%
**Article 35**	Methods	NA	NA	NA	NA	NA	NA
**Article 35**	Results	3/11	6/11	2/11	27%	54%	18%
**Article 35**	Discussion	1/4	0/4	3/4	25%	0%	75%
**Article 35**	Other	0/1	0/1	1/1	0%	0%	100%
**Article 36**	Title	1/1	0/1	0/1	100%	0%	0%
**Article 36**	Abstract	0/1	0/1	1/1	0%	0%	100%
**Article 36**	Introduction	0/2	0/2	2/2	0%	0%	100%
**Article 36**	Methods	4/13	6/13	3/13	30%	46%	23%

		Number of elements reported			Proportion of elements reported		
		No	Unclear/Partial	Yes	No	Unclear/Partial	Yes
**Article 36**	Results	3/11	4/11	4/11	27%	36%	36%
**Article 36**	Discussion	2/4	2/4	0/4	50%	50%	0%
**Article 36**	Other	1/1	0/1	0/1	100%	0%	0%
**Article 37**	Title	NA	NA	NA	NA	NA	NA
**Article 37**	Abstract	0/1	0/1	1/1	0%	0%	100%
**Article 37**	Introduction	0/3	0/3	3/3	0%	0%	100%
**Article 37**	Methods	8/22	6/22	8/22	36%	27%	36%
**Article 37**	Results	1/5	2/5	2/5	20%	40%	40%
**Article 37**	Discussion	0/3	0/3	3/3	0%	0%	100%
**Article 37**	Other	2/4	1/4	1/4	50%	25%	25%
**Article 38**	Title	1/2	1/2	0/2	50%	50%	0%
**Article 38**	Abstract	2/4	2/4	0/4	50%	50%	0%
**Article 38**	Introduction	0/1	0/1	1/1	0%	0%	100%
**Article 38**	Methods	NA	NA	NA	NA	NA	NA
**Article 38**	Results	2/14	3/14	9/14	14%	21%	64%
**Article 38**	Discussion	1/4	1/4	2/4	25%	25%	50%
**Article 38**	Other	1/1	0/1	0/1	100%	0%	0%
**Article 39**	Title	0/1	0/1	1/1	0%	0%	100%
**Article 39**	Abstract	0/1	0/1	1/1	0%	0%	100%
**Article 39**	Introduction	0/2	1/2	1/2	0%	50%	50%
**Article 39**	Methods	7/11	3/11	1/11	63%	27%	9%
**Article 39**	Results	2/8	4/8	2/8	25%	50%	25%
**Article 39**	Discussion	0/4	2/4	2/4	0%	50%	50%
**Article 39**	Other	0/1	0/1	1/1	0%	0%	100%
**Article 19**	Domain 1	0/3	2/3	1/3	0%	66%	33%
**Article 19**	Domain 2	1/3	1/3	1/3	33%	33%	33%
**Article 19**	Domain 3	5/8	3/8	0/8	62%	37%	0%
**Article 19**	Domain 4	1/3	0/3	2/3	33%	0%	66%
**Article 19**	Domain 5	3/4	1/4	0/4	75%	25%	0%
**Article 19**	Domain 6	0/2	0/2	2/2	0%	0%	100%

N/A = Not applicable: abstract only (article 20); CARE reporting guidelines do not require a methods section (articles 15, 26, 35, 38); ARRIVE reporting guideline (article 37) merges title and abstract.

Figure 2. Study quality of 27 research report manuscripts or articles uploaded by the participants in our 2021 medical specialty trainee mandatory research project survey*

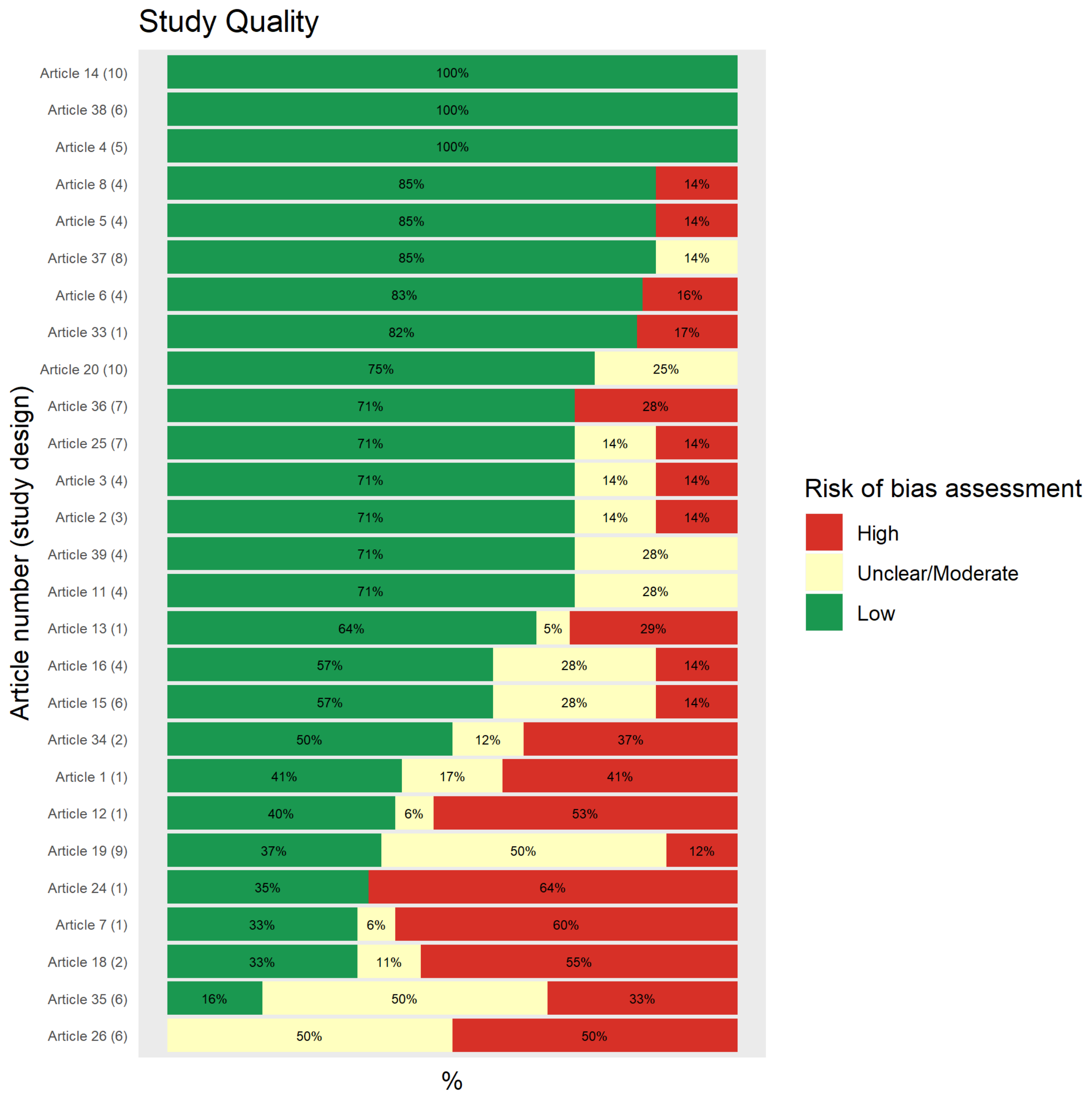

Study design appraisal tools/study types:
Modified AMSTAR 2 (11):
(1) Review - systematic review
(2) Review - scoping review
MMAT (10):
(3) Randomised control trial
(4) Cross sectional
(5) Qualitative
(6) Case series
(7) Cohort with control
(8) Randomised preclinical animal study
Mi-Che (13):
(9) Clinical practice guideline
QADAS 2 (12):
(10) Diagnostic test accuracy study.

Table 7. Calculation of potentially eligible participants

Year	New fellows (TOTAL)	Overseas trained	Eligible
**NEW FELLOWS**			
**2016** (14)	3,262	721	2,541
**2017 (15)**	3,884	272	3,612
**2018 (15)**	3,874	468	3,406
**2019** (15)	3,914	396	3,518
**2020** (15)	3,159	221	2,938
**2021** (15)	4,356	339	4,017
**CURRENT TRAINEES**			
**2021** (15)			23,411
**TOTAL ELIGIBLE**			43,443

Eligible recent fellow calculation: As we were only interested in those who had trained in Australia or New Zealand, eligible recently graduated fellows were calculated by taking the total number of new fellows and taking away those who were overseas trained new fellows. Source: Australian Department of Health and Aged Care. Medical practitioners dashboard [dataset]. https://hwd.health.gov.au/mdcl-dashboards/index.html (viewed Oct 2023).

References

1. Brouwers MC, Kerkvliet K, Spithoff K; AGREE Next Steps Consortium. The AGREE Reporting Checklist: a tool to improve reporting of clinical practice guidelines. BMJ 2016; 352: i1152.
2. Percie du Sert N, Hurst V, Ahluwalia A, et al. The ARRIVE guidelines 2.0: updated guidelines for reporting animal research. BMC Vet Res 2020; 16: 242.
3. Gagnier JJ, Kienle G, Altman DG, et al. The CARE guidelines: consensus-based clinical case reporting guideline development. J Med Case Rep 2013; 7: 223.
4. Dwan K, Li T, Altman DG, Elbourne D. CONSORT 2010 statement: extension to randomised crossover trials. BMJ 2019; 366: l4378.
5. Page MJ, McKenzie JE, Bossuyt PM, et al. The PRISMA 2020 statement: an updated guideline for reporting systematic reviews. PLOS Med 2021; 18: e1003583.
6. Tricco AC, Lillie E, Zarin W, et al. PRISMA Extension for Scoping Reviews (PRISMA-ScR): checklist and explanation. Ann Intern Med 2018; 169: 467-473.
7. O'Brien BC, Harris IB, Beckman TJ, et al. Standards for reporting qualitative research: a synthesis of recommendations. Acad Med. 2014; 89: 1245-1251.
8. Bossuyt PM, Reitsma JB, Bruns DE, et al. STARD 2015: an updated list of essential items for reporting diagnostic accuracy studies. BMJ 2015; 351: h5527.
9. von Elm E, Altman DG, Egger M, et al. Strengthening the Reporting of Observational Studies in Epidemiology (STROBE) statement: guidelines for reporting observational studies. BMJ 2007; 335: 806-808.
10. Hong QN, Fàbregues S, Bartlett G, et al. The Mixed Methods Appraisal Tool (MMAT) version 2018 for information professionals and researchers. Education for Information 2018; 34: 285-291.
11. Shea BJ, Reeves BC, Wells G, et al. AMSTAR 2: a critical appraisal tool for systematic reviews that include randomised or non-randomised studies of healthcare interventions, or both. BMJ 2017; 358: j4008.
12. Whiting PF, Rutjes AW, Westwood ME, et al. QUADAS-2: a revised tool for the quality assessment of diagnostic accuracy studies. Ann Intern Med 2011; 155: 529-536.
13. Siebenhofer A, Semlitsch T, Herborn T, et al. Validation and reliability of a guideline appraisal mini-checklist for daily practice use. BMC Med Res Methodol 2016; 16: 39.
14. National Medical Training Advisory Network. Medical Education and Training in Australia 1st Edition report. In: Department of Health and Aged Care, editor. Australian Government2017. Available from: https://hwd.health.gov.au/resources/publications/report-met1-2016.pdf.
15. Medical Education and Training (MET) 2020 (5th edition) [Dataset]. Department of Heath and Aged Care, Commonwealth of Australia. 2020. Available from: https://hwd.health.gov.au/met-primary/index.html.
